# In Situ Synthetic ZIF-8/Carbon Aerogel Composites as Solid-Phase Microextraction Coating for the Detection of Phthalic Acid Esters in Water Samples

**DOI:** 10.3390/gels8100610

**Published:** 2022-09-25

**Authors:** Zong-Mu Dong, Peiyi Zhang, Tong Sun, Qian Xia, Jian-Feng Wu, Guang-Chao Zhao

**Affiliations:** 1 School of Ecology and Environment, Anhui Normal University, Wuhu 241000, China; 2 Collaborative Innovation Center of Recovery and Reconstruction of Degraded Ecosystem in Wanjiang Basin Co-Founded by Anhui Province and Ministry of Education, Anhui Normal University, Wuhu 241000, China; 3 Anhui Baomei Light Alloy Co., Ltd., Chizhou 242800, China

**Keywords:** carbon aerogel, metal–organic framework, phthalate acid ester, solid-phase microextraction

## Abstract

In this study, a hybrid composite featuring zeolitic imidazolate framework-8/carbon aerogel (ZIF-8/CA) was synthesized via in situ nucleation and growth of ZIF-8 nanoparticles inside carbon aerogels. The novel material was used as the solid-phase microextraction (SPME) coating for the five phthalic acid esters (PAEs) detection by coupling with a gas chromatography–flame ionization detector (GC-FID). Compared with bare carbon aerogel, the ZIF-8/CA presented the best performance, which is attributed to the unique advantages between the high surface area of CA and high hydrophobic properties, the thermal stability of ZIF-8, and their synergistic adsorption effects, such as molecular penetration, hydrogen bond, and π–π stacking interactions. Under the optimized conditions, the as-proposed ZIF-8/CA fiber provided a wide linearity range from 0.2 to 1000 μg L^−1^ and a low detection limit of 0.17–0.48 μg L^−1^ for PAEs analysis. The intra-day and inter-day of signal fiber and the fiber–fiber relative standard deviations were observed in the ranges of 3.50–8.16%, 5.02–10.57%, and 5.66–12.11%, respectively. The method was applied to the determination of five PAEs in plastic bottled and river water samples.

## 1. Introduction

The modified composite carbon aerogel material plays an important role in improving the application of solid-phase microextraction (SPME) and adsorption performance. As far as fiber-based SPME technology is concerned, coating material is the most critical for extraction efficiency and selectivity [1]. Today, some available commercial SPME fiber coating materials such as polydimethylsiloxane (PDMS) and polyacrylate (PA) were used in environmental and food analysis. However, some limitations have also be observed such as poor selectivity for some samples with large polarity differences, poor stability of thermal, and fiber fragility [2,3,4]. To overcome these drawbacks of commercial SPME fibers, a great number of novel excellent materials were developed for improving extraction performance and were applied successfully to detect trace-level organic chemical pollutants in food or environment samples, such as carbon materials [2,5], layered double hydroxides (LDHs) [3], metal–organic frameworks (MOFs) [4,6], and metals/metal oxides [7]. Among these coating materials, MOFs and carbon aerogels materials have attracted considerable research attention due to their fascinating structure and other properties that were appropriate for SPME technology application in these years.

Metal–organic frameworks are new porous crystalline three-dimensional coordination nanoscale polymers formed by metal centers and organic ligands via the self-assemble function process. These materials exhibit permanent nanoscale porosity, high surface area, uniform pore topologies, low densities, various compositions, high adsorption affinity, and good stability of mechanical and thermal (ranging from 200 to 400 °C) [4,6,8,9,10] qualities. Recently, several MOFs have been reported to apply for SPME towards various organic analytes such as UMCM-1 [4], MIL-100(Fe) [6], MIL-88(Fe) [8], and ZIF-8 [9,10]. Among these MOFs, Zeolitic Imidazolate Frameworks (ZIFs) are a typical MOF material with better thermal stability than other MOFs [10]. However, there are still some limitations that need to be solved, such as the low target extraction efficiency and insufficient chemical stability in moisture or the aqueous environment when these materials were applied in SPME [8,9]. To improve the MOFs material properties of chemical stability and extraction efficiency, the main strategies were explored to synthesize some hybrid composites of the MOFs with other stable and efficient nanoscale materials.

Carbon aerogels (CAs) are a network of primary particles with three-dimensional nanostructures with some outstanding properties including a low density, excellent stability, high porosity, high surface area, low cost, and simple preparation [5,11]. As a carbon-based material, CAs exhibited outstanding extraction efficiency for SPME to weak polarity target analytes because of their structure and intrinsic hydrophobicity [2,5,12]. Previous studies have proven that ZIF-8 and CAs can be used as potential applications for SPME fiber coating materials, respectively [5,9]. The CAs could be selected to provide a support in situ growth platform for the typical ZIF-8 crystals. Thus, the composite ZIF-8/CA would provide a better extraction performance for SPME due to the great number of adsorptive sites and multiple chemical function groups from ZIF-8 and the stable crystal platform of CAs with the porous structure. In addition, the existence of Zn^2+^ ions belonging to ZIF-8 has a positive impact on removing hydrophilic groups of CA to enhance the hydrophobicity of composites in the extraction process [13].

Here, by choosing a chemically and thermally robust and highly porous zeolite-type MOF (ZIF-8) as both the precursor with carbon aerogel (CA) as a second precursor and load, the ZIF-8/CA as a new nanomaterial with multifunctional groups, a high specific area, and a large adsorption capacity was successfully prepared by an in situ synthesis method. The ZIF-8/CA composites were then coated onto a prior etched stainless-steel wire to fabricate the SPME fibers. The extraction characteristics of the new fiber were investigated for the extraction of some PAEs as target analytes since these PAEs are widely used in daily life as plasticizers for many personal products such as nail polish, plastic toys for young children, and food bags [14,15,16]. To the best of our knowledge, this is the first report showing the performance of ZIF-8/CA as SPME coatings in complex samples. The main experimental parameters such as the extraction temperature and time, ionic strength, desorption temperature and time, pH value, stirring rate, and sample volume were optimized. The developed method by headspace SPME (HS-SPME) in combination with GC-FID was applied to the extraction of PAEs from river water and bottled pure water samples.

## 2. Results and Discussion

### 2.1. The Selection of the Best Hybrid Ratio of ZIF-8/CA

To confirm the best hybrid ratio between ZIF-8 and CA for SPME application of target analytes, six different mass ratio materials shown in Table S2 were performed and compared the extraction capacity to five PAEs depending on peak area values. The results are shown in Figure 2A. ZIF-8/CA-75 exhibited the best extraction capacity among these materials and composites under the same conditions. As for the reasons why, when the content of ZIF-8 increased continuously, the final composites may lead to a large number of micropores being blocked, the structure of composites may collapse, the density increased, and the pore volume and porosity decreased, according to previous reports [17,18]. A pure ZIF-8-coated fiber with a low extraction capacity may be because of insufficient stability under humid conditions during the extraction process, where the proportion structure of ZIF-8 may be destroyed [8], and the pore size on the surface of the material is too small for PAE molecules to enter the pore cavities in large quantities through the adsorption process. For these reasons, Wt _CA_ = 75% was selected as the best hybrid ratio of ZIF-8/CA for further experiments to obtain a better extraction performance and a more uniform porous structure in the present study.

### 2.2. Characterization of ZIF-8/CA Coating

**Figure 1:**
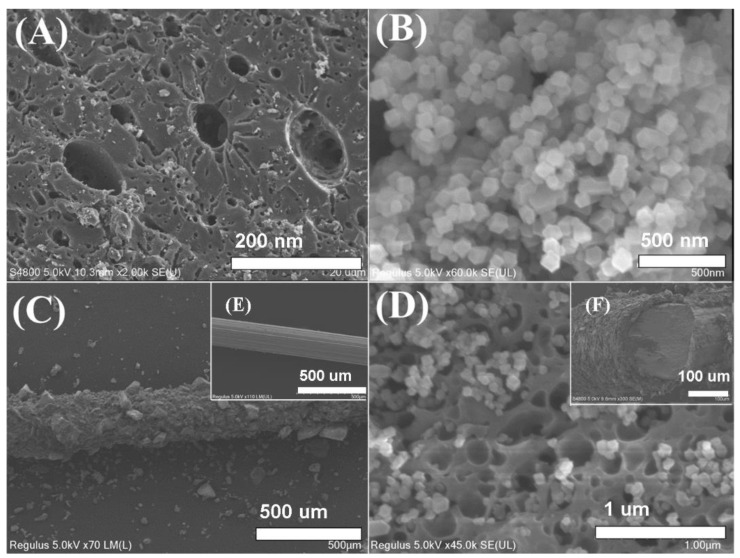
Scanning electron micrograph of (**A**) CA, (**B**) ZIF-8, (**C**) ZIF-8/CA -coated fiber at 70×, and (**D**) ZIF-8/CA coating at 45,000×. In the inner inset, (**E**) naked wire fiber, and (**F**) cross-section of coated fiber.

**Figure 2:**
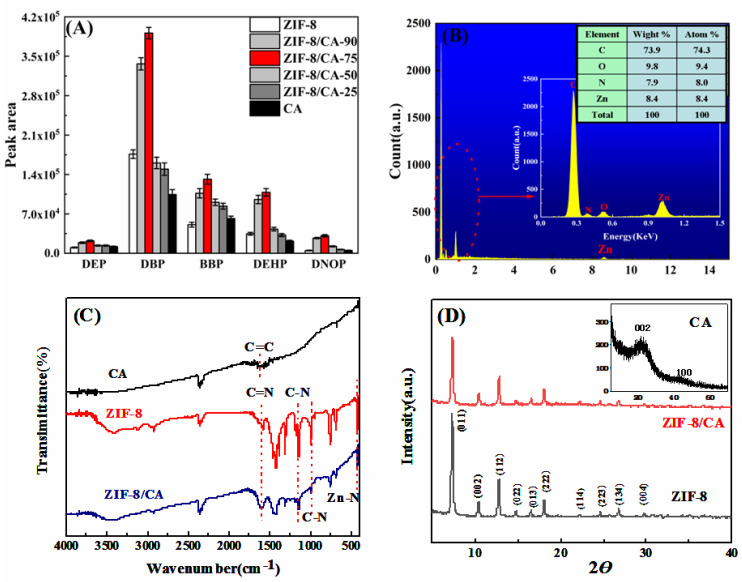
(**A**) Comparison of different mass ratio of composites based on peak area; EDX spectrum (**B**) of ZIF-8/CA; FT-IR patterns (**C**) of CA, ZIF-8, ZIF-8/CA; XRD spectrum (**D**) of ZIF-8, ZIF-8/CA, and CA (the inset).

For the study of the morphological features of the ZIF-8/CA coated fiber, the SEM images of pure CA, ZIF-8, and ZIF-8/CA at different magnifications were recorded. Figure 1A displayed a rich and irregular porous structure network of the CA powders and Figure 1B showed a rhombic structure of the ZIF-8 powders. Furthermore, the low-magnification image in Figure 1C shows that the material was successfully immobilized on the stainless-steel wire and that the ZIF-8/CA coating possessed a relatively homogeneous and rough surface. In the inner inset of Figure 1E the naked steel wire fiber is shown, and Figure 1F shows the cross section of coated fiber, from which it can be observed that the coating thickness is about 25 um. In TEM image (Figure S1B) of the Supplementary Materials, ZF-8 crystal particles with a diameter of 50 nm grow together with CA, and Figure S1A shows the transmission electron microscopy of CA. The high magnification SEM images in Figure 1D further illustrate the morphological structure of ZIF-8/CA, which irregular in situ growth of ZIF-8 crystals in CA pore cavities as support skeleton. In addition, the structure suggests that ZIF-8 crystals had been incorporated into the CA and the expected introduction of N and Zn elements was observed in the fabricated ZIF-8/CA, which has been further verified by an EDX spectrum analysis in Figure 2B. For comparison, the EDX spectrum of CA was also shown in Figure S1D of the Supporting Information.

Figure 2C shows FT-IR spectra of pure CA, ZIF-8, and ZIF-8/CA. For ZIF-8 and ZIF-8/CA, in Figure 2C, corresponding to similar functional group structures, the peaks at about 1591 cm^−1^ can be assigned as the C=N stretch mode, whereas the bands at 1140 and 998 cm^−1^ might be assigned to the C-N group stretching. The bands at 1250–1500 cm^−1^ are associated with the entire imidazole ring stretching, whereas the band below 1250 cm^−1^ is associated with the in-plane bending and out-of-plane bending of imidazole. In addition, the band at 421 cm^−1^ is ascribed to Zn-N stretch [19]. Importantly, pure CA has only one C=C bond with a π-π interaction at 1609 cm^−1^ in Figure 2C (black curve). However, the successful introduction of ZIF-8 adds multiple functional groups with π-π interactions, combining the information of EDX analysis, and the strong p-π conjunction between the N lone pairs and O atoms. The electron cloud of the imidazole rings and CA benzene rings could reduce the density to strengthen the π-π interaction between the rings in ZIF-8/CA material and the benzene rings in target PAE molecules and enhance the affinity of ZIF-8/CA PAEs molecules [20]. These could facilitate the improvement of extraction performance.

The crystalline structure of the ZIF-8/CA was confirmed by comparing the XRD patterns of the synthesized pure ZIF-8 and the ZIF-8/CA. It was found from the XRD diffraction pattern in the inset at the upper right of Figure 2D that the corresponding crystal plane (002) at 2θ = 24°, which is a strong diffraction peak of CA. Furthermore, the pure ZIF-8 and ZIF-8/CA have similar diffraction peaks, such as (011), (222) at 2θ = 7.4° and 18° as shown in Figure 2D, indicating that the existence of CAs does not affect the crystal growth process and formation of metal–organic skeletons. It also proved that the CA material could be the support load flat for ZIF-8 [19]. In addition, the peak intensity of ZIF-8/CA in the XRD is weaker than the pure ZIF-8 sample. The change in peak intensity means that the presence of carbon aerogel affects the crystal orientation of ZIF-8 crystal for some contents, which further confirms the presence of both CA and ZIF-8 in the ZIF-8/CA.

**Figure 3:**
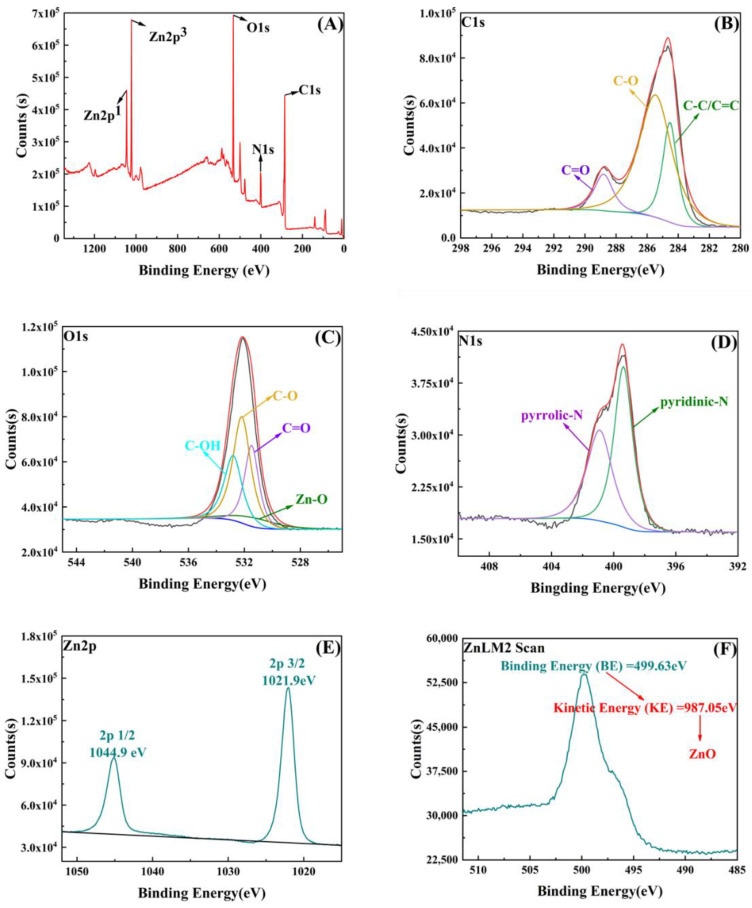
XPS spectra of the survey scan of ZIF-8/CA (**A**), C1s region (**B**), O1s region (**C**), N1s (**D**), Zn2p (**E**) region of ZIF-8/CA, and Auger electron spectrum of Zn (**F**).

To further explore and characterize the chemical bonding composition and mechanism of ZIF-8/CA, multiple regions of the spectrum of ZIF-8/CA were scanned with XPS, including C1s, O1s, N1s, and Zn2p. Meanwhile, an Auger electron spectroscopy (AES) was carried out for the metal element Zn. As displayed in the overall XPS spectrum of ZIF-8/CA in Figure 3A, in which five typical characteristic peaks concentrations of 285.5 eV, 532.2 eV, 400.4 eV, 1022.1 eV and 1045.2 eV are presented, corresponding to C1s, O1s, N1s, Zn2p_1_ and Zn2p_3_, respectively. This further demonstrates that the ZIF-8/CA is composed of four elements: carbon, oxygen, nitrogen, and zinc. The high-resolution spectrum of C1s in Figure 3B shows three peaks at 284.6, 285.5 and 288.8 eV, corresponding to C-C/C=C, C-O and C=O bonds, respectively [21]. Figure 3C, exhibited the O1s spectrum of ZIF-8/CA, which shows four peaks at 530.8 eV, 531.5 eV, 532.2 eV, and 532.8 eV fitted in the experimental spectrum, vesting in Zn-O, C=O, C-O and C-OH, respectively [22,23]. N1s spectrum could be fitted into two peaks in Figure 3D, including pyridinic-N at 399.3 eV, and pyrrolic-N at 400.1 eV [24], which proves the generation of aromatic imidazole rings. As shown in Figure 3E, the Zn2p peak shows a double peak due to the spin-orbit coupling: Zn2p_1/2_ (1044.9 eV) and Zn2p_3/2_ (1021.9 eV) [23], with a binding energy difference of 23.0 eV and S_2p3/2_ and S_2p1/2_ ratio of 2:1. In the meantime, the AES for Zn in Figure 3F was used to explain the valence and form of the Zn element in composite materials, the Binding Energy (BE) of Zn in the composites from the ZnLM2 scan is 499.63 eV, and the X-Ray energy for testing usually is 1486.8 eV. Then, the related Kinetic Energy (KE) was calculated to be 987.05 eV by the data of testing X-Ray energy minus the BE. Furthermore, the presence of Zn in the composite material was determined to be mainly in the form of Zinc oxide (ZnO) by looking at and comparing the XPS datasheet, which demonstrated that the ZIF-8 was combined with CA.

The porous nature data of the ZIF-8/CA, such as surface area and pore distribution, were calculated by using Brunauere–Emmette–Teller (BET) and Barrett–Joyner–Halenda (BJH) model (Figure S2A,B). The BET-specific area of the pure ZIF-8 is 706.925 m^2^ g^−1^ (Figure S2C), the original CA is 218.427 m^2^ g^−1^ (Figure S2D), and the ZIF-8/CA has a surface area of 678.018 m^2^ g^−1^. The CA and ZIF-8 themselves are high specific surface materials, but the composites may be not fully reflected in the BET surface values because of some of the pores being blocked or not detected, and some nanoparticles agglomerated inevitably, then the SBET value of ZIF-8/CA is just between original CA and pure ZIF-8. These data illustrated the successful preparation of composite materials and revealed the ZIF-8 crystals and CA materials may be penetrated partially [17]. In addition, the numerical relationship of the SBET of the three materials in the data in Table S3, found the ZIF-8/CA is slightly smaller than pure ZIF-8, this is similar to reports in the literature [25]. ZIF-8/CA-75 has a suitable pore size and an enhanced BET surface area (678.018 m^2^ g^−1^) among these samples. Thus, ZIF-8 content with a low ratio may facilitate synthesis of the final hybrid composites with a good porous structure morphology and avoid occurring excessive agglomerate. Furthermore, combined with the XRD and FT-IR results, CA and ZIF-8 are more likely a mechanical mixture where the CA and ZIF-8 aggregate together and result in a related, smaller surface area. The results are in good agreement with the previous reported study [26]. In addition, the type IV nitrogen adsorption isotherm with an obvious hysteresis loop was displayed by the ZIF-8/CA sample in Figure S2A, indicating different pore sizes from micro- to meso-pores. Moreover, the specific surface area is not the decisive factor for the extraction efficiency of solid adsorptive materials [27]. Although the specific surface area of ZIF-8/CA is relatively reduced compared to pure ZIF-8 materials, a feasible pore size (3.720 nm) in the pore size distribution curve corresponded to mesopores and pore volume (0.566 m^3^ g^−1^) is obtained, mainly because of the addition of CA, which creates new pores between the ZIF crystals and the CA interface. The pore size of ZIF-8/CA is larger than pure ZIF-8, which is conducive to adsorbing more target analytes. An important point is that the ZIF-8/CA pore size (3.720 nm) is larger than PAEs molecular length [28], which is facilitated to adsorb the PAEs molecular into the mesopores of the prepared ZIF-8/CA material to improve extraction capacity. For these reasons, it could speculate that the composites have an excellent extraction performance for PAEs.

It is important to further consider the thermostability of SPME coating material due to the desorption process conducted inside the GC injector (in an ultrapure N_2_ atmosphere) under high-temperature conditions. Thereby, thermogravimetric analysis (TGA) was used to study the thermostability of ZIF-8/CA and CA materials. As shown in Figure S3A, the as-prepared ZIF-8/CA showed only about 9.7% mass losses up to 300 °C, and the phenolic organic aerogel RFOA showed about 12.6% at the same thermal conditions according to the TGA curve data, indicating the composites have much better thermostability compared with an organic aerogel adsorbent. Therefore, this composite aerogel material is more suitable for gas chromatography.

### 2.3. Optimization of the SPME Methods Using a ZIF-8/CA Coating

To achieve an ideal PAEs extraction efficiency by the ZIF-8/CA coating for HS-SPME, the methods were investigated and optimized for the prepared ZIF-8/CA-coated fibers by a simple factor-by-factor approach. The optimization study was carried out in combination with GC-FID, whereas the extraction efficiency (as peak area) was evaluated for five representative PAEs at 100 μg L^−1^: DEP, DBP, BBP, DEHP, and DNOP. Different parameters were evaluated depending on the SPME mode. The optimal extraction time and temperature were 50 min and 80 °C, and desorption time and temperature were 7 min and 280 °C, respectively. In addition, under the condition of extraction volume of 12 mL, the stirring rate was controlled at 750 rpm, the pH value of sample was controlled at 6, and the salt concentration was selected as 10% NaCl.

#### 2.3.1. Extraction Time

**Figure 4:**
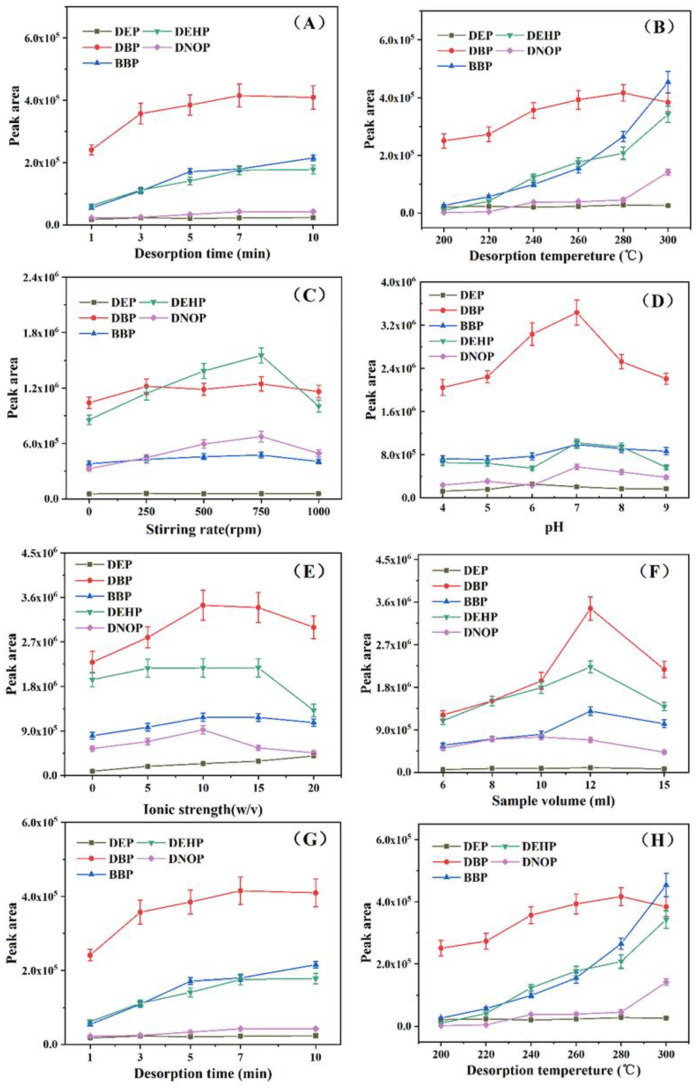
Effect of typical parameters on the peak areas of 100 μg L^−1^ five PAEs. (**A**) Extractant time; (**B**) Extractant temperature; (**C**) Stirring rate; (**D**) pH; (**E**) Ionic strength; (**F**) Sample volume; (**G**) Desorption time; (**H**) Desorption temperature.

SPME is an equilibrium-based pretreatment technique between the amount of analyte to be extracted and the extraction time with an optimum extraction time [3]. In this study, the effect of the extraction time on the extraction of the PAEs was investigated in the range from 20 min to 60 min. Figure 4A shows that the extraction efficiency was first increased from 20 min to 50 min, and then reached a relatively stable stage after 50 min. Therefore, a compromise was made between the extraction time and the amount of analyte extracted, and the optimum extraction time was chosen as 50 min for later experiments.

#### 2.3.2. Extraction Temperature

Temperature is one of the most important parameters that can affect the extraction efficiency in HS-SPME. Altogether, a higher extraction temperature increases the rate of the mass transfer of analytes from the aqueous phase to the headspace phase and hence to the fiber coating, reducing equilibration time and achieving better extraction efficiency [12]. As shown in Figure 4B, the peak areas of the four PAEs showed that the extraction efficiency was increased observably as the extraction temperature was increased from 30 °C to 90 °C. However, considering that a lot of water will evaporate when the extraction temperature is above 90 °C, and good peak area values have been obtained at 80 °C, which can satisfy the requirements of SPME, 80 °C was finally chosen as the best extraction temperature for the later experiments.

#### 2.3.3. Stirring Rate

Effective stirring of the sample solution accelerates the movement of the target analyte molecules and improves the extraction efficiency. The appropriate stirring speed can make the analyte volatilize quickly and reach the gas–liquid equilibrium. However, excessively fast agitation can also lead to the formation of bubbles and vortices in the solution, which can also reduce the stability of the solution, ultimately leading to a decrease in extraction efficiency [9]. As shown in Figure 4C, the extraction efficiency of four PAEs (DBP, BBP, DEHP, and DNOP) was maximal at a stirring rate of 750 rpm. However, the DEP obtained the maximum peak area value at 250 rpm, after which the peak area decreases slightly and stabilizes. Hence, considering the overall situation, the compromise was made by choosing 750 rpm as the most available stirring rate.

#### 2.3.4. pH

Since the ZIF-8 has poor stability under strong acid conditions [29], the pH optimal experiment was performed under related weak acidity and alkalinity conditions (pH from 4 to 9) with as-prepared various pH phosphate buffer salt (PBS) solutions in this study. As shown in Figure 4D, the capacity of fiber gradually decreased in the solution with pH ranging from 4 to 9. Maximum peak area values were obtained at pH 6 for most of the target analytes (DBP, BBP, DEHP, and DNOP), and the other target analyte (DEP) obtained maximum peak area values at pH 7. In order to make the established SPME method achieve a better extraction effect combined with the above experimental results, pH 6 was finally chosen as the best pH value.

#### 2.3.5. Ionic Strength of the Solution

If a certain amount of a salt substance (such as NaCl) is added to an aqueous solution, then the solubility of the organic substance in the aqueous solution will change. The solubility of organic substances in an aqueous solution will be reduced by this “salt-out effect”, and thus the extraction efficiency will be improved [29]. Therefore, the effect of ionic strength on the extraction efficiency was investigated from 0 to 20% (*w*/*v*) NaCl and the results were presented in Figure 4E. The peak area of DEP steadily increased from 0 to 20%. As for the other four analytes, the peak area increased with the increase of ionic strength when the concentration of NaCl was less than 10% (*w*/*v*). Above all, the optimum content of NaCl was selected as 10% (*w*/*v*).

#### 2.3.6. Sample Volume

Different sample volumes were prepared from 6 to 15 mL. As seen in Figure 4F, in the low range of sample volume (6–12 mL), the amount of extraction increased with the increase in volume, and then the increase slowed down. This may be attributed to the fact that SPME is a non-equilibrium extraction process. A large sample volume may not be the only advantage of extraction, and excessive sample volumes can impede the rate of material transfer [30]. The optimal sample volume was selected as 12 mL for future experiments.

#### 2.3.7. Desorption Time

Insufficient desorption time will not only affect the accuracy of the assay results but also the residual analytes on the fiber will affect the next extraction/desorption results. In addition, continuous, high-temperature desorption over a long period time may result in damage to the fibers by fracture [9]. As can be seen from Figure 4G, all analytes were completely desorbed when the desorption process was performed for 7 min. Hence, 7 min was selected as the most suitable desorption time.

#### 2.3.8. Desorption Temperature

Desorption temperature for the SPEM is a key factor to obtain a good sensitivity for the analytes. A higher temperature could facilitate complete desorption of the analyte from coating fiber in the GC injector. However, excessive temperatures could reduce fiber lifespan [8]. In this work, the effect of desorption temperature was investigated by the temperature of the injection port from 200 °C to 300 °C. As shown in Figure 4H, the peak areas of both DEP and DBP decreased slightly when the desorption temperature increased to 280 °C. Therefore, the optimized desorption temperature was controlled at 280 °C in the GC injector.

### 2.4. Method Evaluation

The analytical performance of the ZIF-8/CA-coated fibers in the HS-SPME method was assessed by obtaining the calibration curves in ultrapure water for the group of five PAEs in combination with GC-FID. As listed in Table 1, the ZIF-8/CA-SPME-GC-FID method was validated by determining the linearity, the limit of detection (LOD, signal-to-noise ratio = 3), limit of quantitation (LOQ, signal-to-noise ratio = 10), precision, and reproducibility (relative standard deviation, RSD) under the best conditions. The linear ranges were 0.5–500 μg L^−1^ for BBP and DNOP, 0.5–1000 μg L^−1^ for DEP, and 0.2–1000 μg L^−1^ for DBP under HS-SPME experiments, with a good correlation coefficient (R^2^ ≥ 0.9946), using 12 calibration levels. However, the linear ranges were 1–250 μg L^−1^ for DEHP. It is low, which may be the fact that the ends of two alkane chains in DEHP molecules contain 4 methyl groups. The spatial effect increases the molecular radius and makes it difficult to pass through the pores of the material. The ranges for the LODs and LOQs were 0.17–0.48 μg L^−1^ and 0.58–1.60 μg L^−1^, respectively. The precision of the method was investigated using a single fiber and the results are listed in Table 1. The intra- and inter-day precision ranges, evaluated as RSDs, were 3.50–8.16% and 5.02–10.57%, respectively. The enrichment factor (EF) was calculated and listed in Table 1 through the ratio of analyte which was quantified by the value of peak area after and before extraction (EF = S_SPME_/S_0_). An amount of 100 μg L^−1^ (S_SPME_) of the sample was extracted, and the corresponding S_0_ was obtained via direct injection of 1.0 μL standard solution (100 μg L^−1^) under the same conditions. Three ZIF-8/CA-coated fibers were used to evaluate the fiber-to-fiber reproducibility and the RSD (*n* = 3) range was 5.66–12.11%.

To evaluate the performance of the ZIF-8/CA-SPME-GC-FID method toward PAEs, the developed method was compared with reported methods for the determination of PAEs (Table S4). The present method has acceptable low LODs, and a wider linear range compared with other reported methods [3,4,7,31,32,33,34]. These results reveal that the present method has an excellent extraction performance for PAEs present at trace concentrations and that it is very sensitive and effective compared with other methods.

### 2.5. Analysis of Real Samples

**Figure 5:**
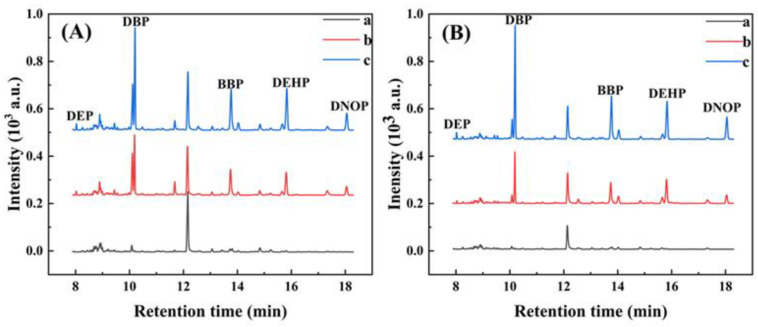
Chromatograms of five PAEs in a real sample. (**A**) River water, (**B**) bottled pure water; (a): blank water; (b): spiked 50 μg L^−1^; (c): spiked 100 μg L^−1^. Peak identity is in order: DEP (1), DBP (2), BBP (3), DEHP (4), DNOP (5). Conditions sample 12 mL; sample pH = 7.0; extraction temperature: 80 °C, time: 50 min; desorption temperature: 280 °C, time: 7 min; stirring rate: 750 rpm.

The ZIF-8/CA-coated fibers were then used for the determination of five PAEs in Huajing River and bottled pure water samples via the SPME process. As listed in Table 2, none of the PAEs was found in the Huajing River or bottled pure water samples and the coating showed a good recovery of PAEs (80.71–117.20%). To better reflect the reproducibility of the experimental analysis method, the recovery rate was calculated by spiking different concentrations of PAEs (50.0, 100.0 μg L^−1^) on two types of real water samples. A typical chromatogram of the Huajing River or bottled pure water samples was shown in Figure 5. Above all that, the ZIF-8/CA exhibited an excellent extraction efficiency, satisfying recovery, and reproducibility by coupling with GC-FID.

### 2.6. Comparison with Other Fibers in Extraction Performance

To evaluate the extraction performance of ZIF-8/CA-coated fibers, several fibers included bare stainless steel (BS), HF-etched stainless steel (HF), epoxy resin-coated stainless steel (ER) and types of different polarity commercial fibers (PDMs, PDMs, Car/PDMs, PA) were used to compared with homemade novel ZIF-8/CA-coated fiber based on peak area values. Moreover, the results were shown in Figure S3B of Supplementary Materials, among these fibers, the ZIF-8/CA-coated fibers showed the outstanding extraction performance and the best extraction capacity except DEP analyte because of the large BET surface, mesopore effect, abundant activated sites of CA and the inherent hydrophobicity, compatible polarity of ZIF-8, strong π-π interactions between the composite materials, and PAEs in this present study.

### 2.7. Durability of Extraction Device

To test the homemade extraction device, which mainly refers to the stability of the novel ZIF-8/CA-coated fiber, different polarity reagents (acetone, methanol, and trichloromethane) and different pH value environments (acidic and alkaline environments) with prepared pH buffer solutions of 6.0 and 8.0 were used to check the chemical stability of the coating material. However, below pH 1, the extraction performance of the coating was found to be only 50% of that under optimal conditions, and the collapse of the coating surface material was observed, which may be attributed to the instability of ZIF-8 under strong acid conditions. The thermal stability was tested by heating the coated fiber under 300 °C in the GC injector for four hours approximately. Briefly, six SPME devices for determination of PAEs (50 μg L^−1^) under the optimal conditions were prepared, then the fiber in these reagent solutions was soaked with a sealing treatment for 12 h, and the above thermal treatment was applied. After each treatment, extraction processes were completed under the same conditions for comparing the obtained peak area values before and after treatments. As shown in Figure S3C, the device based on ZIF-8/CA coating has excellent chemical and thermal stability. In addition, if the artificial SPME devices were reused less than 100 times, the extraction efficiency would not be reduced significantly under the optimal conditions, as in Figure S3D. These results demonstrated that the ZIF-8/CA-SPME-GC-FID method was reliable and very sensitive for determining five PAEs at trace levels.

## 3. Conclusions

In this study, ZIF-8/CA-coated fibers were prepared via a facile in situ synthesis method and employed for the SPME of PAEs in two types of real water samples. Combining the dual advantages of ZIF-8 and CA, the ZIF-8/CA coating clearly showed better performance, including in the abundant functional groups, inherent hydrophobicity, the high specific surface area, and the mesopore effect. The developed method exhibited wide linear ranges, satisfactory low LODs, and good repeatability for the determination of the PAEs from water samples through coupled GC-FID. Thus, the proposed novel ZIF-8/CA coating fibers could be expected to be a promising candidate for SPME coating to detect other residual toxic organic contaminants of water and reasonably other samples at a trace level.

## 4. Materials and Methods

### 4.1. Reagents and Materials

A standard solution (500 μg L^−1^) of five PAEs (DEP, DBP, BBP, DEHP, and DNOP) in dichloromethane was purchased from Bepure (Beijing, China). The structure and some physicochemical properties are in Table S1. A standard stock solution of 100 mg L^−1^ is prepared by diluting five PAEs reagent mixes with acetone and transferring to a 2 mL brown vial to be stored in the refrigerator at 4 °C. Resorcinol (R, 99%) and formaldehyde (F, 37% aqueous solution) were obtained from Aladdin (Shanghai, China). The 2-methylimidazole (miM, 99%) and zinc acetate dihydrate (Zn (NO_3_)_2_·6H_2_O, 99%) were purchased from Aladdin Chemical Reagent Co. Ltd. Deionized water was prepared via an Aquapro water purification system (Chongqing, China). A 50 mm × 0.45 μm cellulose filter membrane was acquired from Lvlong (Nanjin, China). Epoxy resin AB adhesive, stainless steel wire (0.2 mm d.), and plastic-bottled pure water was bought in the local market. Four types of commercial SPME fibers were purchased from Supleco (Sigma-Aldrich, St. Louis, MO, USA). The surface water samples were collected from Huajing River (Wuhu, China). All chemicals and reagents were at least of analytical grade without further purification.

### 4.2. Instruments

A GC-9720 gas chromatograph (Yanzhou, China) equipped with a flame ionization detector (FID) was used to determine PAEs. A 20 m × 0.4 μm with 0.18 mm Rtx-5 GC capillary column (Restek, St. Louis, MO, USA) was used for GC separation. The FID temperature was maintained at 300 °C and the injection was kept in the split-less mode.

A field emission scanning electron microscope S4800 (SEM, Hitachi, Tokyo, Japan) recorded the surface morphology of the as-prepared ZIF-8/CA fiber coating. The morphology, size, and crystal of materials were observed by Transmission electron microscopy (TEM, JEM2100F, Jeol, Tokyo, Japan) at 200 kV. Elemental characterization was conducted by energy-dispersive X-ray (EDX, PHILIPS, S360, and Mv2300). The crystalline phase of samples was identified by X-ray diffraction (XRD, D8 high-resolution Advance Diffractometer, Bruker, Germany) under Cu Ka radiation (45 kV, 30 mA) with a scanning from 5° to 40° at the 5°/min. Fourier transform infrared (FT-IR) spectroscopy was carried out by FT-IR spectrometer 8400S (Shimadzu, Tokyo, Japan), with the scanning from 200 to 4000 cm^−1^. The thermal stability of the obtained coating materials was carried out by thermogravimetric analysis (TGA) using STA 449F5 Jupiter (NETZSCH, Bavaria, Germany) in the range 30–600 °C in N_2_ atmosphere at a 10 °C/min heated rate. X-ray Photoelectron Spectroscopy (XPS, Thermo VG Scientific, Austin, TX, USA) was employed to characterize the chemical composition of the obtained materials by using the Mg Ka X-ray source with a base vacuum operated at 300 W. N_2_ adsorption–desorption isotherms of ZIF-8/CA were obtained using a surface area analyzer BK112T (JWGB SCI & TECH Co., Ltd., Beijing, China).

### 4.3. Preparation of CA

The synthesis of CA was via a modified method process according to a previous report [35]. Briefly, 4.00 g of resorcinol, 3.00 g formaldehyde, and 0.0043 g Na_2_CO_3_ (as a catalyst, C) were dissolved in 15.75 g deionized water (W) at ambient temperature under stirring (molar ratio, R:F:C:W = 1:2:0.008:17.5). After forming a homogeneous solution, the mixture solution was transferred into a sealable polypropylene (PP) container and then completed the aging process in a convection oven. The resulting organic wet gel was cooled to room temperature and immersed into a 10% acetic acid for one day to complete further reaction of organic residues. Before the ambient drying process, solvent substitution was carried out to replace the water (low surface tension solution) in the gel network by putting the organic gel into an acetone (high surface tension solution) bath in a sealed PP container for 3 days. This was followed by drying under ambient conditions for 4 days to obtain monolith dry gel with integrated structure. Finally, the organic aerogels are converted to resultant CA by carbonization in a tubular oven in an ultra-pure nitrogen (>99.999%) atmosphere.

### 4.4. Preparation of ZIF-8/CA

ZIF-8/CA was synthesized by a modified directing in situ growth method at room temperature according to a previous report [26]. In detail, the solutions of 2-methylimidazole (0.15 g mL^−1^) and zinc acetate dehydrate (0.036 g mL^−1^) were mixed and stirred for 30 s to become a homogenous mixture solution. Afterward, the CA as ready-made aerogel templates was added into the above-mixed solution of ligands and central ions based on different mass ratios of CA and aged for 48 h at room temperature to obtain ZIF-8/CA composites. The detailed synthesis information was listed in Table S2. After that growth procedure, the resulting products were rinsed with methanol and deionized water three times, respectively. Finally, the sample was collected by centrifugation at 12,000 rpm for 10 min, and dried at 80 °C for 8 hours in a vacuum oven. Four ZIF-8/CA composites with various CA contents (25%, 50%, 75% and 90%) and one pure ZIF-8 without the addition of CA were synthesized, which were referred to ZIF-8/CA-25, ZIF-8/CA-50, ZIF-8/CA-75, ZIF-8/CA-90, and ZIF-8, respectively.

### 4.5. Preparation of ZIF-8/CA Coated SPME Fiber

Stainless steel wire (200 nm in diameter, 20 cm in length) was used to prepare the ZIF-8/CA-fibers. One end of the stainless steel wire (2.0 cm in length) needs to be roughened via etched in 30% HF for 30 min with an ultrasound situation. Furthermore, the corroded surface was washed with 1mol L^−1^ NaOH solution, ethanol, and deionized water via ultrasonic cleaning for 15 min, respectively. The front of the wire (2.0 cm in length) was dipped into the epoxy resin adhesive solution (dissolved in acetone solution epoxy resin adhesive: acetone = 1:1, *v*/*v*) for 5 s and then rotated in the prepared ZIF-8/CA powders immediately and cured in a convection oven at 100 °C for 30 min. The coating procedures were repeated two times. It was assembled into 10 μL microliter syringes to fabricate the SPME device. The resulting fiber was further aged inside the GC injector at 280 °C with an N_2_ atmosphere until a flat baseline was obtained to reduce possible contaminations and interferences of fiber coating.

### 4.6. HS-SPME Procedure

The HS-SPME process was carried out in a 15 mL headspace amber vial capped with a PTFE-coated septum for all later experiments. The extractions were performed using 12 mL of sample volume diluted with PBS solution (pH = 7.0) at a fixed stirring rate of 750 rpm with an additional 1.2 g NaCl (the ionic strength was adjusted using NaCl as salt addition), and the fiber was exposed to the headspace for 50 min at 75 °C. After the extraction process was completed, the ZIF-8/CA-coated fiber was removed and inserted into the GC for thermal desorption at 280 °C for 7 min for analysis. All the determinations were performed in three replicates.

### 4.7. Real Sample Pretreatment

All water samples were filtered with a 0.45 μm filter membrane to remove all filterable impurities and stored in a suitable size glass vial for the subsequent experiments at 4 °C in refrigerators.

## Figures and Tables

**Table 1:** Analytical performance of the ZIF-8/CA-coated fiber for the SPME of PAEs under the optimized conditions.

Analytes	LR (μg L^−1^)	Regression Equation	R^2^	EF	LODs (μg L^−1^)	LOQs (μg L^−1^)	RSDs (%)		
							One Fiber Inter-Day (*n* = 5)	One Fiber Intra-Day (*n* = 5)	Fiber to Fiber (*n* = 3)
DEP	0.5–1000	y = 1350.2x + 1420.4	0.9985	252	0.32	1.05	5.97	3.74	5.66
DBP	0.2–500	y = 16,117x + 82,578	0.9996	642	0.17	0.58	5.02	7.06	9.50
BBP	0.5–1000	y = 5768.1x + 102,527	0.9959	329	0.22	0.72	10.57	5.71	12.11
DEHP	1–250	y = 6136.4x + 36,030	0.9991	544	0.48	1.60	7.14	3.50	8.48
DNOP	0.5–500	y = 2141.6x + 22,282	0.9946	641	0.30	0.99	7.02	8.16	9.13

**Table 2:** Analytical results for the determination of five PAEs compounds in Huajing River and bottled water samples.

Analytes	River Water Samples				Bottled Water Samples			
	Spiking (μg L^−1^)	Found (μg L^−1^)	Recovery (%)	RSD (%, *n* = 3)	Spiking (μg L^−1^)	Found (μg L^−1^)	Recovery (%)	RSD (%, *n* = 3)
DEP	0	ND *	-	-	0	ND	-	-
	50	42.21	84.42	4.98	50	42.27	84.54	3.66
	100	80.71	80.71	10.97	100	83.83	83.83	3.24
DBP	0	ND	-	-	0	ND	-	-
	50	40.58	81.16	12.31	50	40.41	80.82	7.70
	100	83.41	83.41	7.31	100	81.14	81.14	9.32
BBP	0	ND	-	-	0	ND	-	-
	50	52.76	105.52	9.71	50	56.79	113.58	4.00
	100	117.20	117.20	10.63	100	97.97	97.97	4.22
DEHP	0	ND	-	-	0	ND	-	-
	50	52.80	105.60	11.54	50	52.65	105.30	4.07
	100	108.28	108.28	11.62	100	102.24	102.24	8.65
DNOP	0	ND	-	-	0	ND	-	-
	50	58.57	117.14	5.81	50	56.25	112.50	8.13
	100	114.47	114.47	4.00	100	117.50	117.50	15.30

* ND: not detected.

## Data Availability

Not applicable.

## Supplementary Materials

The following supporting information can be downloaded at: https://www.mdpi.com/article/10.3390/gels8100610/s1. Figure S1: TEM images of (A) CA, (B) ZIF-8/CA, and EDX images of (C) CA, (D) ZIF-8/CA (The inner table shows the content of each element). Figure S2: N_2_ adsorption–desorption isotherm (A) and BJH adsorption pore volume–pore diameter distribution curve (B) of ZIF-8/CA, N_2_ adsorption–desorption isotherm of pure ZIF-8 (C) and original CA (D). Figure S3: (A) TGA curves of original CA and ZIF-8/CA; (B) Effect of eight fibers on extraction efficiency; (C) The stability of the coating tested in different organic solvents (acetone, trichloromethane, and methanol), different pH (pH = 6.0, 8.0), and high-temperature (300 °C); (D) Comparison of the extraction performance of the ZIF-8/CA-coated fiber after different cycles of extraction/desorption. Table S1: Structure and some physicochemical properties of PAEs; Table S2: The compositions of the synthesis mass ratio of the product; Table S3: Pore structure parameters of the sample; Table S3: Pore structure parameters of the sample; Table S4: Comparison of the established method with the other reported methods for detection PAEs.
