# Supplementary material for: In Situ Synthetic ZIF-8/Carbon Aerogel Composites as Solid-Phase Microextraction Coating for the Detection of Phthalic Acid Esters in Water Samples
Source: Gels. 2022 Sep 25;8(10):610. doi: 10.3390/gels8100610 (PMC9602289; doi:10.3390/gels8100610)
Supplement: Supplementary file 1 [file gels-08-00610-s001.zip › gels-1902256-supplementary.pdf]

Supplementary Materials

In-situ synthetic ZIF-8/carbon aerogel composites as solid-phase microextraction coating for the detection of phthalic acid esters in water samples

Zong-Mu Dong[1, 2] *, *Peiyi Zhang*[1], *Tong Sun*[1, 3], *Qian Xia*[1], *Jianfeng Wu*[1, 2], *Guang-Chao Zhao*[1]*

1. School of Ecology and Environment, Anhui Normal University, Wuhu 241000, China

2, Collaborative Innovation Center of Recovery and Reconstruction of Degraded Ecosystem in Wanjiang Basin Co-founded by Anhui Province and Ministry of Education, Anhui Normal University, Wuhu 241000, China

3. Anhui Baomei Light Alloy Co., Ltd., Chizhou 242800, China

***Corresponding author**: Zong-Mu Dong: dzongmu@mail.ahnu.edu.cn; Guang-Chao Zhao: gczhao@mail.ahnu.edu.cn

Tel: +86 553 5910724; Fax: +86 553 5910720

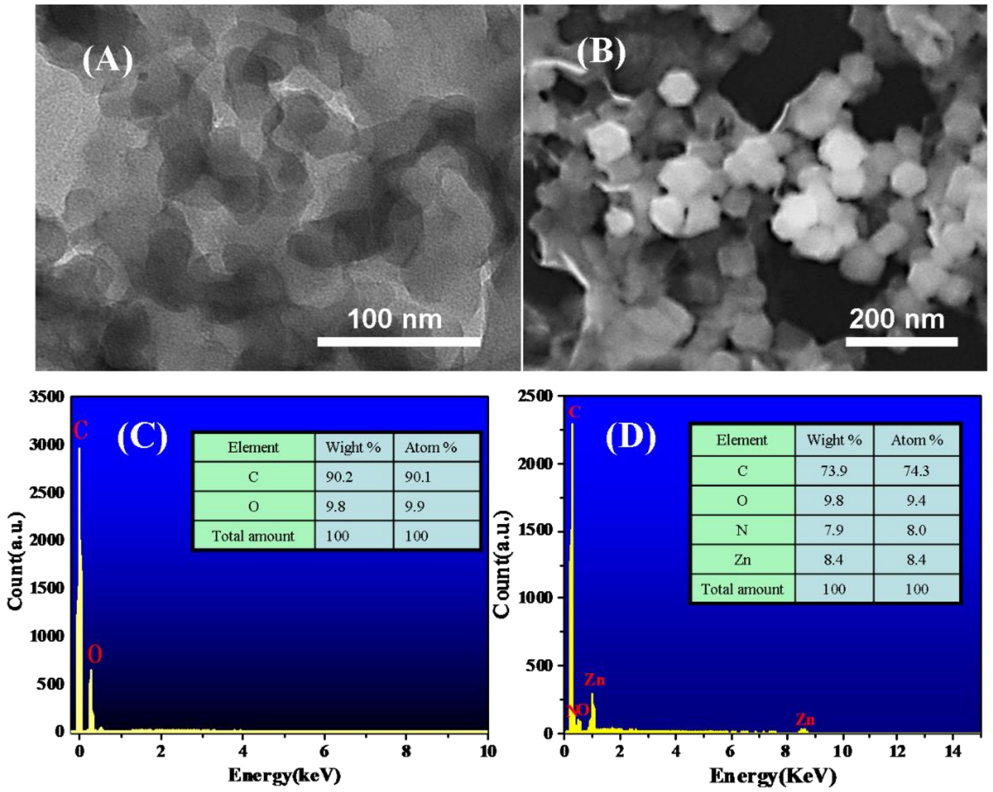

Figure S1 TEM images of (A) CA, (B) ZIF-8/CA, and EDX images of (C) CA, (D) ZIF-8/CA (The inner table shows the content of each element)

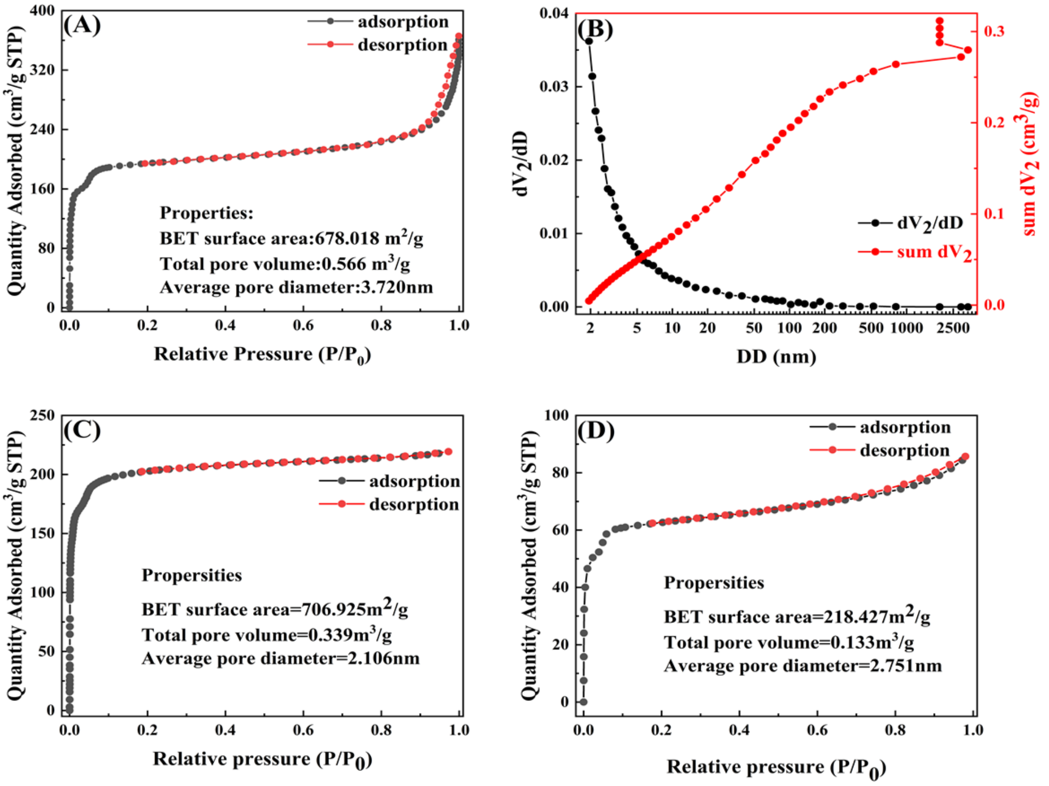

Figure S2. N_2 adsorption–desorption isotherm (A) and BJH adsorption pore volume–pore diameter distribution curve (B) of ZIF-8/CA, N_2 adsorption–desorption isotherm of pure ZIF-8 (C) and original CA (D).

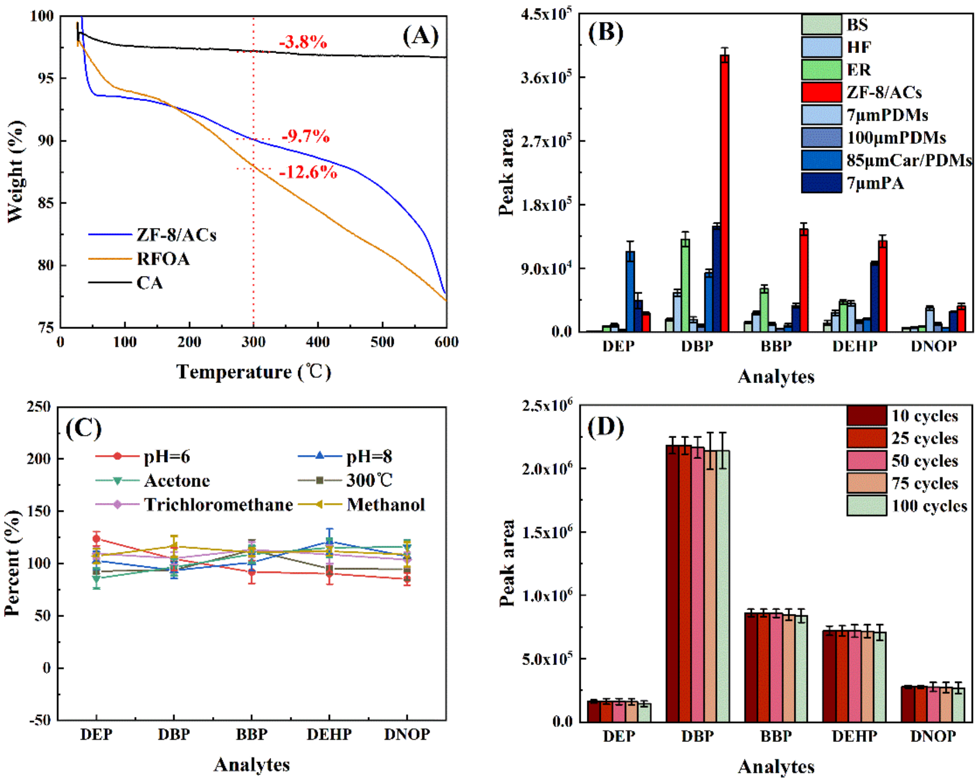

Figure S3. (A) TGA curves of original CA and ZIF-8/CA; (B) Effect of eight fibers on extraction efficiency; (C) The stability of the coating tested in different organic solvents (acetone, trichloromethane, methanol), different pH (pH=6.0, 8.0) and high-temperature (300 ℃); (D) Comparison of the extraction performance of the ZIF-8/CA -coated fiber after different cycles of extraction/desorption. Conditions of (A) and (B): sample volume, 10 mL; extraction time, 30 min; extraction temperature, 60 ℃; desorption time, 5min; desorption temperature, 260℃; no additional salt; no stirring; no pH adjustment; sample level, 100 μg L^{-1}.Conditions of (C) and (D): extraction time, 50 min; extraction temperature, 80℃; desorption time, 7min; desorption temperature, 280℃; stirring rate, 750 rpm; ionic strength, 10%; pH, 6.0; sample volume, 12 mL; PAEs concentration, 100 μg L^{-1}.

Table S1 Structure and some physicochemical properties of PAEs.

[a]Data comes from ChemBK website

Target analyte	Structural formula	CAS. No.	Molecular formula	Molecular weight	Boiling point	K_{ow}[a]
DEP	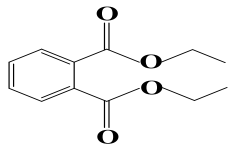	84-66-2	$C_{12}H_{14}O_4$	222.24	298℃	295.0
DBP	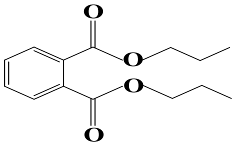	84-74-2	$C_{16}H_{22}O_4$	278.34	340℃	3.5×10^5
BBP	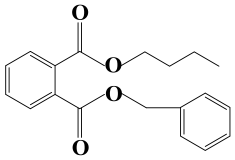	85-68-7	$C_{19}H_{20}O_4$	312.36	370℃	7.4×10^9
DEHP	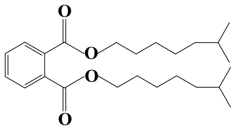	117-81-7	$C_{24}H_{38}O_4$	390.56	386℃	4.1×10^9
DNOP	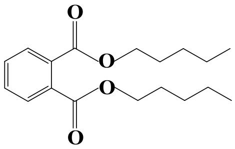	117-84-0	$C_{24}H_{38}O_4$	390.56	380℃	3.6×10^5

a. N-octanol-water partition coefficient

(https://www.chembk.com/cn)

Table S2. The compositions of the synthesis mass ratio of the product.

Sample	CA (g)	mIM (g)	Zn $(NO_3)_2$ $6H_2O$ (g)	Production (g)
ZIF-8	0	15	3.6	0.18
ZIF-8/CA -90	1.82	15	3.6	2.00
ZIF-8/CA -75	0.54	15	3.6	0.72
ZIF-8/CA -50	0.18	15	3.6	0.36
ZIF-8/CA -25	0.06	15	3.6	0.24

Table S3 Pore structure parameters of the sample.

Sample	S_{BET}[a](m^2/g)	V_{total}[b](m^3/g)	$D_{average}$[c] (nm)
ZIF-8	706.93	0.34	2.11
ZIF-8/CA -90	319.26	0.35	4.92
ZIF-8/CA -75	678.02	0.57	3.72
ZIF-8/CA -50	230.51	0.20	3.73
ZIF-8/CA -25	196.08	0.16	3.71
CA	218.43	0.13	2.75

a. BET specific surface areas

b. Total pore volume,

c. Average pore diameter

Table S4 Comparison of the established method with the other reported methods for detection PAEs.

**Analytical method**	**Sorbent/ extractant**	**LR ($\mu g\ L^{-1}$)**	**LODs ($\mu g\ L^{-1}$)**	**Analytes**	**Application**	**Ref.**
SPME-GC-FID	Mg/Al-LDH-H_2O_2	1-500	0.42-1.29	DBP, BBP, DEHP, DNOP	River water and bottled mineral water	[1]
UA-DLLME-SFOD-GC-FID [a]	n-hexadecane	5.03-536	0.64-2.57	DBP, BBP, DCHP, DEHP, DNOP	Water, wine, vinegar and soft drink	[2]
SPME-GC-FID	$CuFe_2O_4$ NPs [b]	1-500	0.12-0.40	DBP, BBP, DEHP, DNOP	Bottled mineral water	[3]
HFLMP-SPME-GC-FID [c]	UMCM-1 MOF	0.01-1000	0.008-0.03	DMP, DEP, DIBP, DNBP	Yogurt, water and edible oil	[4]
MISPE-GC-MS [d]	MIP [e]	5-100	13.00-22.00	DMP, DEP, DBP, DAP, DNOP	Milk	[5]
SPME-GC-FID	G/PVC [f]	0.45-100	100-150	DPP, DBP, DEHA, DEHP	Drinking water and edible oil	[6]
SPME-GC-μECD	PA6 [g]-MnO NPs	0.5-500	0.021-0.3	DMP, DEP, DBP, DEHP	River water, bottled water, mineral water and soda drink	[7]
SPME-GC-FID	ZCACs	0.2-1000	0.17-0.48	DEP, DBP, BBP, DEHP, DNOP	River water and bottled pure water	This study

a. ultrasound-assisted dispersive liquid–liquid microextraction
b. copper ferrite nanoparticles
c. hollow fiber liquid membrane-protected solid-phase microextraction
d. molecularly imprinted solid-phase extraction-gas chromatograph and mass spectrometry
e. Molecularly imprinted polymer
f. graphene/polyvinylchloride
g. polyamide 6

References

[1] Tian, T.; Wang, F.; Zhao, G. Magnesium/aluminum-layered double hydroxide modified with hydrogen peroxide as a novel fiber coating for solid-phase microextraction of phthalate esters in aqueous samples. *Microchem. J.* **2020**, 153, 104510.

[2] Pérez-Outeiral, J.; Millán, E.; Garcia-Arrona, R. Determination of phthalates in food simulants and liquid samples using ultrasound-assisted dispersive liquid–liquid microextraction followed by solidification of

floating organic drop. *Food Control* **2016**, 62, 171-177.

[3] Wu, D.; Liu, F.; Tian, T.; Wu, J.; Zhao, G. Copper ferrite nanoparticles as novel coating appropriated to solid-phase microextraction of phthalate esters from aqueous matrices. *Microchem. J.* **2021**, 162, 105845.

[4] Mirzajani, R.; Kardani, F.; Ramezani, Z. Fabrication of UMCM-1 based monolithic and hollow fiber-Metal-organic framework deep eutectic solvents/molecularly imprinted polymers and their use in solid phase microextraction of phthalate esters in yogurt, water and edible oil by GC-FID. *Food Chem.* **2020**, 314, 126179.

[5] He, J.; Lv, R.; Zhu, J.; Lu, K. Selective solid-phase extraction of dibutyl phthalate from soybean milk using molecular imprinted polymers, *Anal. Chim. Acta* **2010**, 661, 215-221.

[6] Amanzadeh, H.; Yamini, Y.; Moradi, M.; Asl, Y. Determination of phthalate esters in drinking water and edible vegetable oil samples by headspace solid phase microextraction using graphene/polyvinylchloride nanocomposite coated fiber coupled to gas chromatography-flame ionization detector. *J. Chromatogr. A* **2016**, 1465, 38-46.

[7] Eskandarpour, N.; Sereshti, H. Electrospun polycaprolactam-manganese oxide fiber for headspace-solid phase microextraction of phthalate esters in water samples. *Chemosphere* **2018**, 191, 36-43.
